# Effects of short-term moderate intensity exercise on the serum metabolome in older adults: a pilot randomized controlled trial

**DOI:** 10.1038/s43856-024-00507-w

**Published:** 2024-05-04

**Authors:** Jie Jun Wong, Jien Sze Ho, Louis L. Y. Teo, Hai Ning Wee, Kee Voon Chua, Jianhong Ching, Fei Gao, Swee Yaw Tan, Ru-San Tan, Jean-Paul Kovalik, Angela S. Koh

**Affiliations:** 1 National Heart Centre Singapore, Singapore, Singapore; 2 Duke-NUS Medical School, Singapore, Singapore; 3 Singapore General Hospital, Singapore, Singapore

**Keywords:** Heart failure, Metabolomics

## Abstract

**Background:**

We previously reported changes in the serum metabolome associated with impaired myocardial relaxation in an asymptomatic older community cohort. In this prospective parallel-group randomized control pilot trial, we subjected community adults without cardiovascular disease to exercise intervention and evaluated the effects on serum metabolomics.

**Methods:**

Between February 2019 to November 2019, thirty (83% females) middle-aged adults (53 ± 4 years) were randomized with sex stratification to either twelve weeks of moderate-intensity exercise training (Intervention) (*n* = 15) or Control (*n* = 15). The Intervention group underwent once-weekly aerobic and strength training sessions for 60 min each in a dedicated cardiac exercise laboratory for twelve weeks (ClinicalTrials.gov: NCT03617653). Serial measurements were taken pre- and post-intervention, including serum sampling for metabolomic analyses.

**Results:**

Twenty-nine adults completed the study (Intervention *n* = 14; Control *n* = 15). Long-chain acylcarnitine C20:2-OH/C18:2-DC was reduced in the Intervention group by a magnitude of 0.714 but increased in the Control group by a magnitude of 1.742 (mean difference −1.028 age-adjusted *p* = 0.004). Among Controls, alanine correlated with left ventricular mass index (r = 0.529, age-adjusted *p* = 0.018) while aspartate correlated with Lateral e’ (r = −764, age-adjusted *p* = 0.016). C20:3 correlated with E/e’ ratio fold-change in the Intervention group (r = −0.653, age-adjusted *p* = 0.004). Among Controls, C20:2/C18:2 (r = 0.795, age-adjusted *p* = 0.005) and C20:2-OH/C18:2-DC fold-change (r = 0.742, age-adjusted *p* = 0.030) correlated with change in E/A ratio.

**Conclusions:**

Corresponding relationships between serum metabolites and cardiac function in response to exercise intervention provided pilot observations. Future investigations into cellular fuel oxidation or central carbon metabolism pathways that jointly impact the heart and related metabolic systems may be critical in preventive trials.

## Plain language summary

Prior studies have found changes in cellular biological processes in both cardiac aging and heart failure suggesting a common underlying mechanism. I has also been shown that exercise in healthy participants can reverse the signs of early cardiac aging. In this experimental study, we examined the effects of exercise on biological markers and cardiac function among healthy community older adults. After twelve weeks of exercise, there were changes in biological components associated with cardiac function. These findings highlight the potential of exercise as a strategy to target biological alterations in early cardiac aging and potentially prevent it.

## Abstract (web summary)

Wong et al. conduct a pilot randomized controlled trial on the effects of twelve weeks of exercise on the serum metabolome in older adults. The authors identify changes in metabolites related to mitochondrial fuel oxidation and amino acid pathways and relate these to imaging markers of cardiac aging in post-hoc analyses.

## Introduction

Age-related alterations in the cardiovascular system, such as ventricular and arterial stiffening, contribute to the risks of developing heart failure in aging^1^. Aging is commonly associated with changes in myocardial systolic and diastolic function, although diastolic dysfunction is a predominant feature that frequently precedes symptomatic systolic dysfunction^1–4^. Diastolic dysfunction also plays an important role in the pathophysiology of overt clinical heart failure with preserved ejection fraction (HFpEF), a subset of heart failure of substantial prevalence in aging cohorts^5,6^. While the underlying pathophysiological mechanisms behind diastolic dysfunction are complex, impairments in mitochondrial oxidation and fuel metabolism pathways have delineated diastolic failure from systolic failure^7,8^. These pathways were similarly observed among aging, asymptomatic older adults with impairments in myocardial relaxation and mitochondrial fuel metabolism^9,10^. Thus, understanding early metabolic changes in aging-related ventricular stiffness may reveal mechanistic insights before the onset of clinical diastolic failure.

The aging heart is associated with a decline in mitochondrial content and function with knock-on effects on major fuel metabolism pathways^11,12^. Recent evidence has linked the accumulation of amino acids such as leucine and long-chain acyl-carnitines with early impaired myocardial relaxation and arterial stiffness in an aging population^9,13^. Metabolomic studies in HFpEF have also revealed similar associations between disease and accumulation of long-chain acyl-carnitines^14,15^. Thus, in both cardiovascular aging and HFpEF, changes in markers of fuel metabolism link dysfunctional mitochondrial metabolism and central carbon pathways to the development of cardiovascular disease^9,16^. Given these similar functional and metabolic changes for cardiovascular aging and HFpEF, we hypothesize that interventions targeting these common pathways in the early stages may represent novel strategies applicable to aging-related diastolic dysfunction as an early upstream approach.

Aging-related changes in cardiac compliance are accelerated by physical inactivity and can be partially reversed by exercise^17^. Increases in long-chain acyl-carnitines have been linked to lower aerobic capacity^18^, while exercise upregulates mitochondrial and related fuel metabolism pathways in the heart and can reverse aging-related decline in skeletal muscle mitochondrial function^19,20^. Increased physical activity upregulates fatty oxidation and Krebs cycle genes, activating central carbon metabolism and mitochondrial pathways^21^. These findings suggest that exercise may reverse the metabolic underpinnings of cardiac aging and potentially prevent early aging-related diastolic dysfunction.

Given the importance of exercise as an essential modifiable lifestyle factor in mitigating aging-related cardiovascular risk, a better understanding of the effect of physical activity on underlying cellular metabolic processes will improve our understanding of disease pathophysiology and highlight new potential targets for disease prevention. In this paper, we examine the effects of exercise on human metabolism in the early stages of healthy aging that can be detected using metabolomic signatures. We additionally explore the associations between serum metabolomic markers in relation to cardiac function and hemodynamic parameters.

## Methods

### Study population

**Fig. 1:**
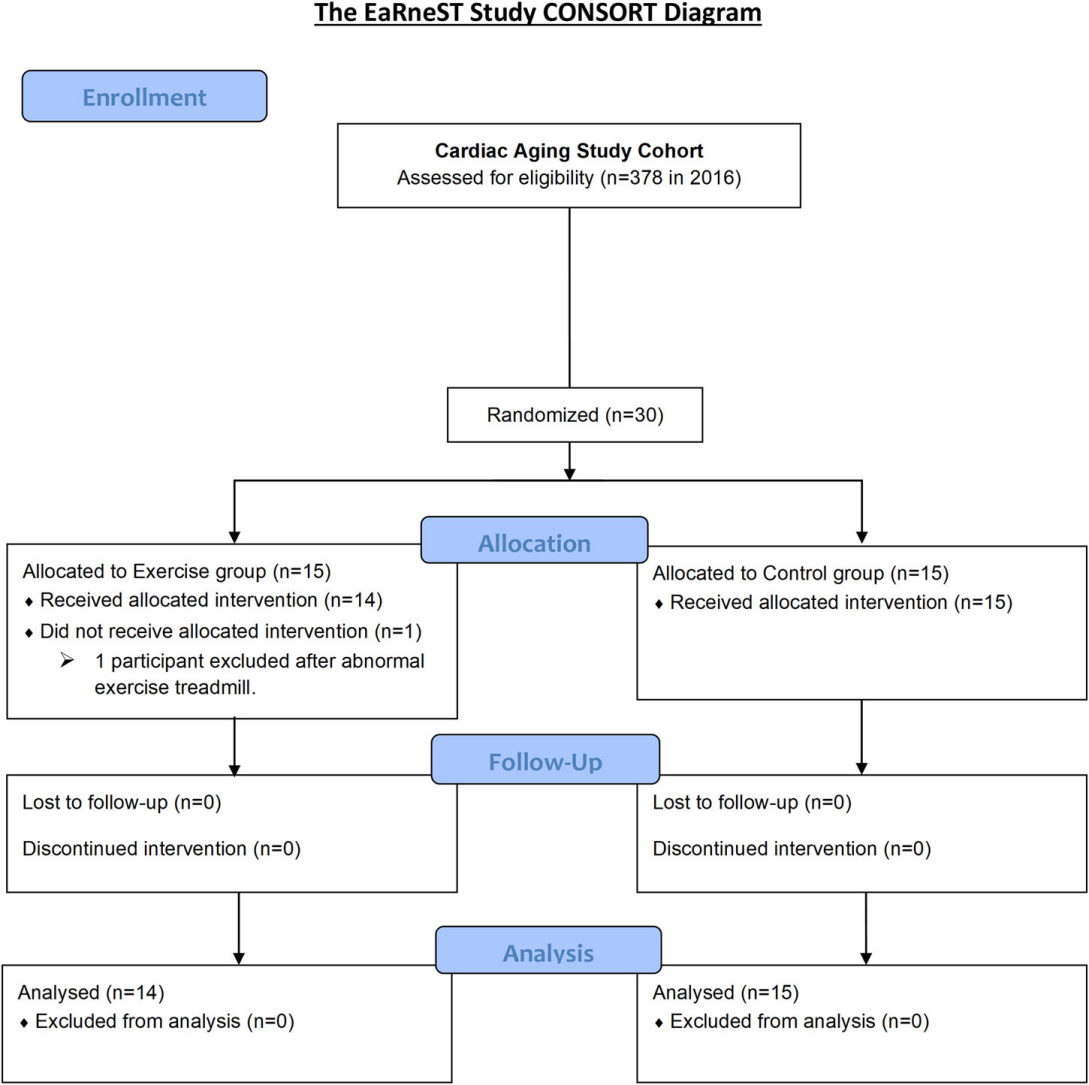
The EaRneST trial CONSORT flow diagram. Thirty community adults from the Cardiac Aging Study had volunteered to participate in this exercise trial between February 2019 and November 2019 and were randomized by sex-stratification equally into Intervention (Exercise) and Control groups. Within the Intervention group, one participant was excluded after an abnormal exercise treadmill test, while the remaining 14 participants completed the exercise intervention. In total, 29 participants were analysed at the study completion.

Community adults with no prior evidence of cardiovascular disease underwent transthoracic echocardiography as part of the Cardiac Aging Study, a prospective study that examines characteristics and determinants of cardiac structural and functional changes at age^18^. Thirty participants volunteered to participate in this exercise trial between February 2019 and November 2019 (Fig. 1). Participants were recruited if they were 40 and above, were willing and able to provide informed consent, and had at least grade 1 diastolic dysfunction on echocardiography based on the latest guidelines^22^. Participants with a history of cardiovascular disease, stroke, cancer, uncontrolled hypertension (i.e., systolic blood pressure ≥160 mmHg and/or diastolic blood pressure ≥ 90 mmHg despite being on treatment for hypertension), low blood pressure (i.e., systolic blood pressure < 90 mmHg or diastolic blood pressure < 40 mmHg), acute pulmonary embolus or pulmonary infarction, acute myocarditis or pericarditis, suspected or known dissecting aneurysm, acute systemic infection, or uncontrolled metabolic disease (e.g., diabetes, thyrotoxicosis or myxoedema) were excluded. Patients with neuromuscular, musculoskeletal, or rheumatoid disorders that are exacerbated by exercise were also excluded. Written informed consent was obtained from participants upon enrolment. The study was conducted in accordance with the Declaration of Helsinki, and the SingHealth Centralized Institutional Review Board (CIRB/2018/2118) approved the study protocol. They were randomized with sex stratification into either twelve weeks of exercise training (Intervention, *n* = 15) or no exercise (Control, *n* = 15). Before exercise intervention, participants underwent a treadmill stress test to exclude undiagnosed obstructive coronary artery disease. One participant with an equivocal treadmill test result was excluded from the study. Treadmill test results were read by independent board-certified cardiologists blinded to participation details.

### Data acquisition

Participants underwent baseline measurements before intervention and repeated measurements after 12 weeks since randomization. These measurements include anthropometry, body composition, central hemodynamics, transthoracic echocardiogram, and serum sampling for metabolomic analyses.

Skeletal muscle mass was measured by a multifrequency bioimpedance body composition analyzer (InBody 370, Biospace Company). Appendicular lean mass (ALM) was calculated as the sum of muscles for the bilateral upper and lower limbs, and ALM/height^2^ was calculated as follows: ALM (kg)/height (m^2^), respectively.

Central blood pressure and pulse wave analysis were measured with the Schiller BR-102 PLUS pulse wave analysis device (Schiller AG, Baar, Switzerland) using the oscillometric method on the brachial artery. Brachial diastolic and systolic blood pressure values were obtained from the portable automatic blood pressure function. Blood pressure was recorded multiple times during the day. Each participant was seated comfortably in a quiet room before blood pressure was recorded^23^. A single examiner performed all measurements. The pulse wave analysis was based on auscultatory/oscillometric blood pressure measurement and pulse contour analysis, providing information about arterial behavior and pulse wave velocity. The following parameters were obtained: (1) aortic augmentation index (%) with and without correction for a heart rate of 75 (AIx@HR75), (2) pulse wave velocity (m/s), (3) peripheral resistance (s*mmHg/ml), (4) estimated central systolic and diastolic pressure and (5) central pulse pressure calculated as the difference between estimated systolic and diastolic BP.

Two-dimensional transthoracic echocardiography was performed using ALOKA α10 with a 3.5 MHz probe. In each subject, standard echocardiography, which included 2-D, M-mode, pulse Doppler, and tissue Doppler imaging, was performed in the standard parasternal and apical (apical 4-chamber, apical 2-chamber, and apical long) views, and three cardiac cycles were recorded. Left ventricular ejection fraction and left atrial volume were measured. The trans-mitral flow E and A wave with the sample volume position at the tip of the mitral valve leaflets from the apical 4-chamber view were recorded by Doppler echocardiography. The E/A ratio was computed as a ratio of peak velocity flow in early diastole E (m/s) to peak velocity flow in late diastole by atrial contraction A (m/s). Pulsed wave tissue Doppler imaging was performed with the sample volume at the septal and lateral annulus from the apical 4-chamber view. The frame rate was between 80 and 100 frames per second. The tissue velocity patterns were recorded and expressed as E’ and A.’ Stroke volume (SV) was calculated using the Doppler volume time integral (VTI) method by multiplying left ventricular outflow tract VTI with its cross-sectional area^24,25^. Cardiac output was calculated by multiplying SV with pulse rate. Total arterial compliance was estimated using the SV/PP method by dividing stroke volume with pulse pressure (PP)^26,27^. Effective arterial elastance was calculated using the Pes/SV method by dividing the LV end-systolic pressure (Pes) by stroke volume^28^. Echocardiograms were performed by ultrasonographers blinded to the allocation of groups and interpreted by independent board-certified cardiologists blinded to participation details. Blood samples were collected on the day of echocardiography acquisition. Urinary microalbumin was measured using standard assays.

### Metabolomics profiling

Antecubital venous blood samples (20–30 ml) were taken from consenting participants in the morning; fasting was not required before blood collection. After collection, the blood samples were immediately placed on ice for transportation and were processed within six hours to obtain serum samples. Serum metabolomic profiling analysis was performed in the Duke-NUS Metabolomics Facility. Serum samples (50 μl) were spiked with a 10 μl deuterium-labeled amino acid mixture and diluted with 400 µl methanol. After centrifugation of the mixture at 17,000 g for 5 min at 4 °C, the supernatant fraction was collected (10 μl) for amino acid analysis. A pooled quality control (QC) sample was prepared by mixing equal amounts (10 μl) of each extracted serum sample. Extraction and measurement of amino acid panels (quantified in units of μM) were performed as previously described in ref. ^10^. The methanol extracts were derivatized with 3 M Hydrochloric acid in butanol (Sigma Aldrich, USA) for amino acid analysis and diluted in water for analysis in LC-MS. For amino acid analysis, a C18 column (Phenomenex, 100 × 2.1 mm, 1.6 μm, Luna® Omega) on an Agilent 1290 Infinity LC system (Agilent Technologies, CA, USA) coupled with quadrupole-ion trap mass spectrometer (QTRAP 5500, AB Sciex, DC, USA) were used. Mobile phase A (Water) and Mobile phase B (Acetonitrile) containing 0.1% Formic acid were used for chromatography separation. The LC run was performed at a flow rate of 0.4 mL min^−1^ with an initial gradient of 2% B for 0.8 min, then increased to 15% B in 0.1 min, 20% B in 5.7 min, 50% B in 0.5 min, 70% B in 0.5 min, followed by re-equilibration of the column to the initial run condition (2% B) for 0.9 min. All compounds were ionized in positive mode using electrospray ionization. The chromatograms were integrated using MultiQuant™ 3.0.3 software (AB Sciex, DC, USA). For acyl-carnitines, serum samples (100 μl) were spiked with a 20 μl deuterium-labeled acyl-carnitine mixture and diluted with 800 µl methanol. Extraction and measurement of acyl-carnitine were performed as previously described^9^. Measurements were taken from distinct samples. Data acquisition and analysis were performed on an Agilent MassHunter Workstation B.06.00 Software.

### Exercise Intervention

The exercise group received once-weekly exercise sessions for twelve sessions. Each weekly session consisted of aerobic exercise on treadmill machines lasting 20 –30 min (at a moderate pace of 75–85% peak heart rate), followed by strength training of major muscle groups (shoulders, chest, arms, and legs) lasting 20 min, with warm-up and cool-down using simple stretches and gentle aerobics movements lasting 10 min each.

Exercise physiologists met with participants personally throughout the intervention. Exercise sessions were conducted in person. Exercise physiologists directly supervised the exercise sessions and reviewed the participant’s progress and adherence to the intervention. No adverse effects were recorded in this trial.

Participants randomized to the Control group were advised not to participate in outside formal exercise training programs. They were allowed to do their leisure exercise.

### Study endpoints

The study’s primary outcome measure was the change in metabolomic markers after 12 weeks of exercise intervention. Post-hoc analyses were carried out to examine associations between metabolomic markers, echocardiographic left ventricle indices, and vascular compliance at baseline and with respect to their change over time.

### Statistics and Reproducibility

Continuous variables are expressed as mean (standard deviation), and categorical variables as n (%). Baseline and post-intervention characteristics between the groups were compared using Fisher’s exact tests for categorical variables and Mann-Whitney U test for continuous variables. Wilcoxon signed-rank test was used for matched pre- and post-intervention comparisons within the groups. Fold-change was calculated by dividing post-intervention values with pre-intervention values. Spearman’s rank-order correlation and scatter plots were used to examine the associations between the changes in metabolites, echocardiography, and vascular compliance. Subsequently, for outcomes between groups with *p* < 0.050, multiple regression was further applied to adjust for clinical characteristics that were different between the groups (i.e., age). For metabolites, analytes with > 10% outlier values were excluded. Outliers were defined as values below the limit of detection for the analyte. Missingness was replaced by the lowest detected value for that analyte. These metabolites are usually of low abundance and uncertain biological importance. Metabolites were standardized during laboratory analysis^9,10^. Log-transformation and scaling were not performed. Statistical analyses were done using IBM SPSS Statistics for Windows, Version 23 (IBM Corp, Armonk, NY, USA).

### Reporting summary

Further information on research design is available in the Nature Portfolio Reporting Summary linked to this article.

## Results

### Participant characteristics

Thirty participants were randomized by sex stratification into Control (54.8 years ±3.55, males 13.3%) and Intervention groups (51.4 years ±4.34, males 20%). The Intervention group was younger by 3.2 years on average (*p* = 0.037); otherwise, both groups exhibited similar clinical characteristics, including body mass index, waist circumference, blood pressure, and maximal oxygen uptake (Table 1). In total, 29 participants completed the twelve-week study, with 14 participants in the Intervention group and 15 in the Control group. All Intervention group participants completed twelve sessions of exercise. There were no deviations from the pre-specified trial protocol.

**Table 1:** Baseline characteristics

Variables	Control group (*n* = 15)	Exercise training group (*n* = 15)	*p*-value
Age, y	54.8 (3.6)	51.4 (4.3)	0.037
Male, *n* (%)	2 (13.3%)	3 (20.0%)	0.082
Weight, kg	58.9 (8.6)	62.4 (11.6)	0.436
Height, cm	157.2 (7.3)	162.0 (8.2)	0.067
Body mass index, kg/m^2^	23.9 (3.4)	23.6 (2.8)	1.000
Hypertension	0 (0%)	0 (0%)	-
Dyslipidaemia	0 (0%)	1 (6.7%)	0.309
Diabetes mellitus	0 (0%)	0 (0%)	-
Peripheral Systolic blood pressure, mmHg	122.5 (18.6)	121.9 (13.0)	0.683
Peripheral Diastolic blood pressure, mmHg	70.7 (12.0)	71.8 (12.5)	0.967
Pulse rate, per minute	72.5 (9.4)	70.3 (11.8)	0.250
VO^2^ max, mL/kg/min	35.3 (5.0)	36.5 (4.7)	0.461

Values are shown as mean (standard deviation) or n (%). VO2max indicates maximal oxygen uptake. Mann Whitney U tests were used for comparisons. *P*-values are two-tailed.

### Effect of exercise intervention: Changes in Metabolomics

**Fig. 2:**
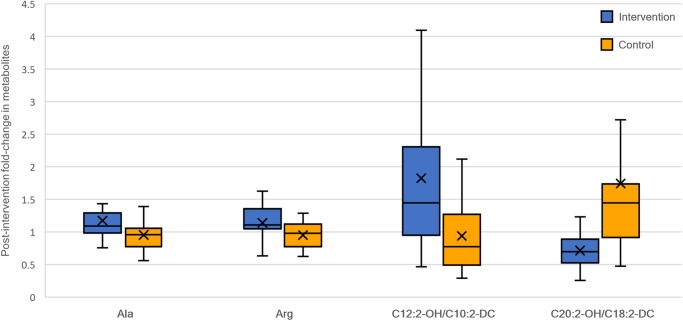
Effect of intervention on metabolomic markers. Box-and-whisker plots of fold-change in serum metabolomics after the exercise period in the Intervention (*n* = 14) and Control (*n* = 15) groups. The horizontal lines represent quartiles, while the cross (denoted “X”) represents the mean value. The error bars represent the range from the minimum and maximum values. Mann Whitney U tests were used for comparisons. *P*-values are two-tailed. In the Intervention group, there were significant relative increases in serum amino acids alanine (*p* = 0.029) and arginine (*p* = 0.046), as well as C12:2-OH/C10:2-DC (*p* = 0.033) compared to Controls, whilst C20:2-OH/C18:2-DC decreased (*p* < 0.001). Ala indicates alanine, Arg Arginine.

We evaluated the change in metabolomic markers after 12 weeks of exercise intervention as the primary outcome measure. There were accumulations of medium-chain acylcarnitine C12:2-OH/C10:2-DC in the Intervention group by a magnitude of 1.825 (fold-change), with corresponding reductions in the Control group by a magnitude of 0.9376 (mean difference in fold-change 0.888, *p* = 0.033, age-adjusted *p* = 0.070) (Table 2). Levels of long-chain acylcarnitine C20:2-OH/C18:2-DC were reduced in the Intervention group by a magnitude of 0.7136 (fold-change) but increased in the Control group by a magnitude of 1.741 (mean difference in fold-change 1.028, *p* < 0.001, age-adjusted *p* = 0.004). Alanine (fold-change 1.1733 vs. 0.95344, mean difference 0.220, *p* = 0.029, age-adjusted *p* = 0.070) and arginine (fold-change 1.1379 vs. 0.9501, mean difference 0.188 *p* = 0.046, age-adjusted *p* = 0.133) were both increased in the Intervention group and decreased in the Control group (see Fig. 2). Adjusting for age, only the changes in C20:2-OH/C18:2-DC persisted. A heatmap of serum metabolites after the intervention is shown in Supplementary Fig. 1.

**Table 2:** Fold Changes in Metabolomics

Metabolite	Control mean (*n* = 15)	Intervention mean (*n* = 14)	Mean Difference	*p*-value	Age-adjusted *p*-value	95% CI
C2	1.24	1.16	−0.08	0.533		−0.35 to −0.20
C3	0.92	1.09	0.17	0.683		−0.15 to −0.48
C4	1.02	1.07	0.05	0.983		−0.19 to −0.28
C5:1	1.48	0.93	−0.55	0.880		−1.52 to −0.43
C5	1.03	1.06	0.03	0.505		−0.28 to −0.33
C4-OH	1.34	2.02	0.68	0.252		−1.10 to −2.46
C6	1.28	1.24	−0.04	0.715		−0.64 to −0.56
C5-OH/C3-DC	1.02	1.02	0.004	0.914		−0.32 to −0.33
C4-DC,C6-OH	1.35	1.14	−0.21	0.880		−0.71 to −0.29
C8:1	1.24	1.41	0.17	0.505		−0.45 to −0.79
C8	1.69	2.06	0.37	0.533		−0.70 to −1.45
C5-DC	1.17	1.20	0.04	0.747		−0.28 to −0.35
C8:1-OH/C6:1-DC	1.39	1.34	−0.05	0.949		−0.53 to −0.43
C8-OH/C6-DC	1.32	1.46	0.14	0.780		−0.45 to −0.73
C10:3	1.34	1.48	0.14	0.747		−0.56 to −0.84
C10	1.77	2.02	0.25	0.621		−0.86 to −1.36
C7-DC	1.21	1.24	0.03	0.652		−0.35 to −0.41
C8:1-DC	1.07	1.54	0.48	0.290		−0.08 to −1.04
C8-DC	1.73	1.59	−0.14	0.813		−1.26 to −0.98
C12:2	1.30	1.79	0.49	0.234		−0.35 to −1.33
C12:1	1.47	1.73	0.26	0.425		−0.55 to −1.08
C12	1.72	1.57	−0.14	0.983		−1.04 to −0.76
C12:2-OH/C10:2-DC	0.94	1.83	0.89	0.033	0.070	0.09 to −1.68
C12:1-OH	1.19	1.85	0.67	0.123		−0.56 to −1.89
C12-OH/C10-DC	1.78	1.43	−0.34	0.949		−1.17 to −0.48
C14:3	1.40	2.15	0.75	0.505		−0.65 to −2.14
C14:2	1.57	1.62	0.05	0.983		−0.68 to −0.78
C14:1	1.53	1.68	0.15	0.683		−0.72 to −1.03
C14	1.49	1.19	−0.30	0.505		−0.86 to − 0.27
C14:3-OH/C12:3-DC	2.85	4.00	1.15	0.451		−2.41 to −4.72
C14:2-OH	1.43	1.60	0.17	1.000		−0.94 to −1.27
C14:1-OH	1.22	1.20	−0.02	0.983		−0.44 to −0.40
C14-OH/C12-DC	1.62	1.37	−0.25	0.813		−0.98 to −0.48
C16:3	1.33	1.81	0.48	0.186		−0.34 to −1.31
C16:2	1.76	1.52	−0.25	0.747		−1.33 to −0.84
C16:1	1.42	1.30	−0.11	0.747		−0.68 to −0.46
C16	1.16	1.02	−0.14	0.234		−0.38 to −0.11
C16:3-OH/C14:3-DC	1.21	1.63	0.42	0.780		−0.64 to −1.47
C16:2-OH	1.37	2.31	0.94	0.533		−0.99 to −2.87
C16:1-OH/C14:1-DC	1.42	1.15	−0.27	0.477		−0.76 to −0.22
C16-OH	1.002	1.14	0.14	0.505		−0.20 to −0.46
C18:3	1.27	1.51	0.24	0.880		−0.58 to −1.06
C18:2	0.97	0.98	0.01	0.683		−0.20 to −0.22
C18:1	1.13	1.02	−0.10	0.505		−0.38 to −0.17
C18	1.17	1.05	−0.12	0.377		−0.39 to −0.14
C18:3-OH/C16:3-DC	1.17	1.27	0.10	0.561		−0.30 to −0.51
C18:2-OH/C16:2-DC	1.31	1.26	−0.05	0.914		−0.65 to −0.54
C18:1-OH/C16:1-DC	1.63	1.54	−0.09	0.451		−1.32 to −1.13
C18-OH/C16-DC	1.22	1.52	0.29	0.949		−0.54 to −1.13
C20:4	1.05	1.02	−0.04	0.949		−0.39 to −0.32
C20:3	0.94	1.27	0.34	0.561		−0.23 to −0.90
C20:2	1.10	0.96	−0.15	0.715		−0.5 to −0.21
C20:1	1.03	1.24	0.22	0.591		−0.17 to −0.60
C20	1.11	1.19	0.08	0.983		−0.31 to −0.46
C20:3-OH/C18:3-DC	0.97	2.02	1.06	0.400		−0.66 to −2.77
C20:2-OH/C18:2-DC	1.74	0.71	−1.03	< 0.001	0.004	−1.80 to −0.26
C20:1-OH/C18:1-DC	1.41	1.23	−0.18	0.683		−0.69 to −0.32
C20-OH/C18-DC	1.24	1.18	−0.06	0.880		−0.45 to −0.34
C22:5	1.36	1.54	0.18	0.683		−0.56 to −0.93
C22:3	1.20	1.74	0.54	0.715		−0.71 to −1.79
C22:2	1.65	2.18	0.52	0.310		−1.23 to −2.28
C22:1	1.02	1.27	0.25	0.112		−0.55 to −1.06
C22	1.09	1.22	0.13	0.621		−0.41 to −0.67
C24	1.26	1.17	−0.09	0.949		−0.64 to −0.47
C26	1.15	1.17	0.02	0.561		−0.33 to −0.38
C28	1.61	1.49	−0.12	0.186		−1.54 to −1.29
Glycine	0.99	1.09	0.10	0.217		−0.02 to −0.22
Alanine	0.95	1.17	0.22	0.029	0.070	0.01 to −0.43
Serine	0.97	1.07	0.10	0.270		−0.08 to −0.27
Proline	0.91	1.07	0.16	0.252		−0.07 to −0.39
Valine	1.01	1.04	0.03	1.000		−0.14 to −0.2
Leucine	0.99	1.05	0.05	0.505		−0.20 to −0.3
Isoleucine	1.06	0.998	−0.06	0.914		−0.36 to −0.24
Ornithine	0.90	0.92	0.02	0.425		−0.15 to −0.18
Methionine	0.97	1.14	0.18	0.310		−0.11 to −0.46
Histidine	0.94	0.99	0.06	0.290		−0.04 to −0.16
Phenylalanine	0.97	0.99	0.02	0.914		−0.12 to −0.15
Arginine	0.95	1.14	0.19	0.046	0.133	0.01 to −0.37
Citrate	0.91	1.04	0.14	0.102		−0.04 to −0.32
Tyrosine	0.98	1.12	0.14	0.112		−0.07 to −0.35
Asparagine	0.85	0.84	−0.01	0.813		−0.19 to −0.17
Glutamate	0.92	0.91	−0.01	0.683		−0.15 to −0.13
Tryptophan	0.96	1.06	0.10	0.063		−0.08 to −0.29

Values are shown as mean. *P*-values are two-tailed. Significant *p*-values (< 0.050) are further adjusted for age using multiple regression. Measurements were taken from distinct samples.

### Effect of exercise intervention: cardiovascular parameters

We then performed post-hoc analyses on the associations between metabolomic markers, echocardiographic markers, and hemodynamic parameters at baseline and with respect to their change over time.

### Hemodynamics and arterial stiffness

In the Control group, central diastolic blood pressure by 8.34 mmHg (*p* = 0.036), and central systolic blood pressure increased by 13.5 mmHg (*p* = 0.028); there was a trend towards increased pulse wave velocity by 0.986 m/s (*p* = 0.059) which did not achieve statistical significance (Table 3). Comparing both groups post-intervention, the Intervention group had a lower pulse wave velocity by 0.70 m/s (*p* = 0.036), which was not significant after adjusting for age (*p* = 0.485) (see Table 4). We did not observe significant changes in the total arterial compliance index between the groups (Table 4 and Supplementary Table 1).

**Table 3:** Pre- and Post-Intervention Hemodynamics, Cardiovascular function, and Associated Markers

	Control group (*n* = 15)			Exercise training group (*n* = 14)		
	Pre	Post	*p*-value	Pre	Post	*p*-value
Peripheral Systolic blood pressure, mmHg	122.5 (18.6)	122.9 (19.2)	0.861	121.9 (13.0)	118.2 (14.5)	0.551
Peripheral Diastolic blood pressure, mmHg	70.7 (12.0)	77.0 (13.9)	0.063	71.8 (12.5)	74.1 (11.8)	0.683
Heart Rate, beats per minute	72.5 (9.4)	69.9 (13.7)	0.256	69.2 (11.4)	65.8 (15.4)	0.221
Pulse wave velocity, m/s	7.13 (0.86)	8.12 (0.64)	0.059	7.73 (1.56)	7.42 (0.79)	0.575
AIX@75	27.6 (10.5)	30.5 (12.5)	0.683	26.6 (4.5)	33.3 (9.4)	0.123
Central systolic blood pressure, mmHg	103.2 (9.2)	116.7 (15.5)	0.028	108.8 (9.8)	114.7 (15.6)	0.327
Central diastolic blood pressure, mmHg	73.4 (5.4)	81.75 (11.7)	0.036	75.2 (9.1)	79.4 (8.5)	0.624
Central Pulse Pressure, mmHg	29.8 (6.4)	34.9 (9.3)	0.541	33.6 (8.7)	35.3 (11.9)	0.483
Weight, kg	58.9 (8.6)	59.6 (9.30)	0.334	62.4 (11.6)	62.3 (12.06)	0.683
LA volume, ml	32.7 (11.1)	38.4 (8.9)	0.244	38.9 (7.1)	40.4 (6.8)	1.000
LA volume index, ml/m^2^	20.4 (6.8)	23.8 (4.8)	0.307	23.5 (5.3)	24.4 (4.2)	0.975
Septal e’, m/s	0.09 (0.02)	0.09 (0.01)	0.209	0.09 (0.02)	0.09 (0.02)	0.220
Lateral e’, m/s	0.120 (0.026)	0.108 (0.019)	0.033	0.108 (0.017)	0.110 (0.021)	0.462
E/e’ ratio	7.55 (1.40)	7.73 (1.21)	0.470	7.96 (1.70)	7.74 (1.61)	0.975
E/A ratio	1.44 (0.43)	1.34 (0.27)	0.495	1.31 (0.30)	1.39 (0.41)	0.975
Cardiac index, L/min^2^	2913.4 (654.1)	2857.3 (627.5)	0.733	2602.4 (283.8)	2754.3 (551.9)	0.730
Stroke index, mL/m^2^	41.1 (8.0)	41.2 (6.8)	0.910	40.5 (5.2)	41.9 (5.8)	0.583
Total arterial compliance index, mL/mmHg m^2^	0.85 (0.29)	0.92 (0.18)	0.307	0.82 (0.36)	0.94 (0.40)	0.152
Effective arterial elastance index, mL/mmHg m^2^	1.77 (0.59)	1.71 (0.35)	1.000	1.64 (0.25)	1.54 (0.22)	0.422
Skeletal Muscle Mass, kg	21.0 (3.2)	20.9 (3.3)	0.509	22.4 (5.4)	22.3 (5.9)	0.682
Basal Metabolic Rate, Cal/day	1206.4 (115.6)	1211.9 (117.4)	0.755	1264.1 (192.9)	1261.9 (214.3)	0.950
Appendicular Lean Mass, kg	15.6 (2.7)	15.7 (2.8)	0.460	17.1 (4.3)	16.9 (4.8)	0.363
6MWT, m	510.9 (106.9)	562.4 (79.7)	0.182	511.9 (111.2)	523.5 (90.8)	0.972
Urine Albumin:Creatinine ratio	1.43 (1.00)	4.01 (5.81)	0.433	1.39 (0.92)	2.02 (1.67)	0.362

Values are shown as mean (standard deviation). Wilcoxon signed-rank tests were used for paired comparisons. *P*-values are two-tailed. 6MWT indicates six-minute walk test; AIX@75, Augmentation Index corrected for heart rate; E/A ratio, ratio of early to late peak diastolic mitral inflow velocity; E/e’ ratio, ratio of early peak mitral inflow velocity to peak early diastolic mitral annular velocity; Lateral e’, Peak early diastolic lateral mitral annular velocity; Septal e’, Peak early diastolic septal mitral annular velocity.

**Table 4:** Post-intervention Hemodynamics, Cardiovascular function, and Associated Markers

	Control (*n* = 15)	Intervention (*n* = 14)	*p*-value	Adjusted *p*-value
Peripheral Systolic blood pressure, mmHg	122.9 (19.2)	118.2 (14.5)	0.652	
Peripheral Diastolic blood pressure, mmHg	77.0 (13.9)	74.1 (11.8)	0.813	
Heart rate, beats per minute	69.9 (13.7)	65.8 (15.4)	0.108	
Pulse wave velocity, m/s	8.12 (0.64)	7.42 (0.79)	0.036	0.485
AIX@75	30.5 (12.5)	33.3 (9.4)	0.674	
Central systolic blood pressure, mmHg	116.7 (15.5)	114.7 (15.6)	0.974	
Central diastolic blood pressure, mmHg	81.8 (11.7)	79.4 (8.5)	0.628	
Central pulse pressure, mmHg	34.9 (9.3)	35.3 (11.9)	0.722	
Weight, kg	59.6 (9.30)	62.3 (12.06)	0.631	
LA volume, ml	38.4 (8.9)	40.4 (6.7)	0.505	
LA volume index, ml/m^2^	23.8 (4.8)	24.4 (4.2)	0.880	
Septal e’, m/s	0.086 (0.013)	0.091 (0.016)	0.354	
Lateral e’, m/s	0.108 (0.019)	0.110 (0.021)	0.667	
E/e’ ratio	7.73 (1.21)	7.74 (1.61)	0.804	
E/e’ ratio fold-change	1.06 (0.26)	1.01 (0.26)	0.804	
E/A ratio	1.34 (0.27)	1.39 (0.41)	0.591	
E/A ratio fold-change	0.97 (0.23)	1.06 (0.29)	0.451	
Cardiac index, L/min^2^	2857.3 (627.5)	2754.3 (551.9)	0.683	
Stroke index, mL/m^2^	41.2 (6.8)	41.9 (5.8)	0.618	
Total arterial compliance index, mL/mmHg m^2^	0.92 (0.18)	0.937 (0.40)	0.525	
Effective arterial elastance index, mL/mmHg m^2^	1.71 (0.35)	1.54 (0.22)	0.088	
Skeletal Muscle Mass, kg	20.9 (3.3)	22.3 (5.9)	1.000	
Basal Metabolic Rate, Cal/day	1211.9 (117.4)	1261.9 (214.3)	1.000	
Appendicular Lean Mass, kg	15.7 (2.8)	16.9 (4.8)	0.983	
6MWT, m	562.4 (79.7)	523.5 (90.8)	0.252	
Urine Albumin:Creatinine ratio	4.0 (5.8)	2.02 (1.7)	0.949	

Values are shown as mean (standard deviation). Mann Whitney U tests were used for comparisons. *P*-values are two-tailed. Significant *p*-values (< 0.050) are further adjusted for age. 6MWT indicates six-minute walk test; AIX@75, augmentation Index corrected for heart rate; E/A ratio, ratio of early to late peak mitral inflow velocity; E/e’ ratio, ratio of early peak mitral inflow velocity to early peak diastolic mitral annular velocity; Lateral e’, peak early diastolic lateral mitral annular velocity; Septal e’, peak early diastolic septal mitral annular velocity.

### Left ventricular diastolic and systolic function

**Fig. 3:**
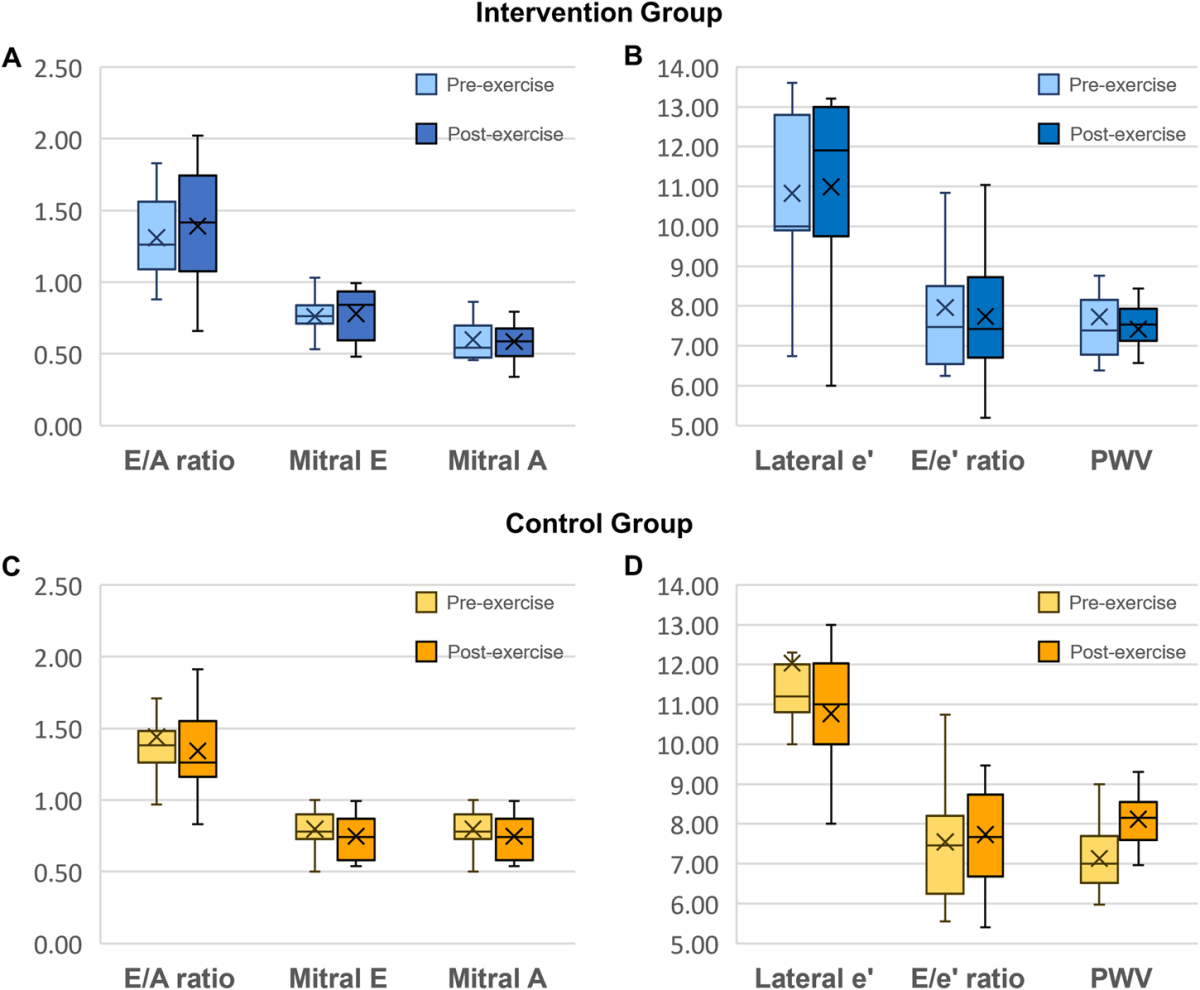
Effect of intervention on diastolic function and vascular stiffness. Box-and-whisker plots before and after exercise intervention. The horizontal lines represent quartiles, while the cross (denoted “X”) represents the mean value. The error bars represent the range from the minimum and maximum value. **A**, **B** Changes in echocardiographic parameters and pulse wave velocity after the exercise period in the Intervention group (*n* = 14). **C**, **D** Changes in echocardiographic parameters and pulse wave velocity in the Control group (*n* = 15). The units for Lateral e’ are reported here in cm/second to fit the y-axis of the other variables. Error bars represent quartiles with the mean marked “X”. Mann Whitney U tests were used for comparisons. *P*-values are two-tailed. In the Control group, Lateral e' decreased (*p* = 0.033), and pulse wave velocity increased (*p* = 0.059). The Intervention group remained unchanged. Mitral A wave indicates mitral inflow peak velocity in late diastole; E wave, early diastolic mitral inflow peak velocity; lateral e’, lateral mitral annular peak velocity in early diastolic; E/e’, ratio of mitral E wave to the average e’.

In the Intervention group, Lateral e’ increased by 0.10 cm/s (*p* = 0.462, age-adjusted *p* = 0.003). In the Control group, Lateral e’ decreased by 1.3 cm/s (*p* = 0.033, age-adjusted *p* = 0.585) but was not significant after age adjustment (Table 3, Supplementary Table 2). Changes in E/e’ ratio or E/A ratio were non-significant. (Table 4 and Fig. 3a–d). We did not observe significant changes in left ventricular ejection fraction, cardiac index, or stroke volume index between the groups (Table 4 and Supplementary Table 1).

### Correlations between metabolomics and echocardiographic indices

**Fig. 4:**
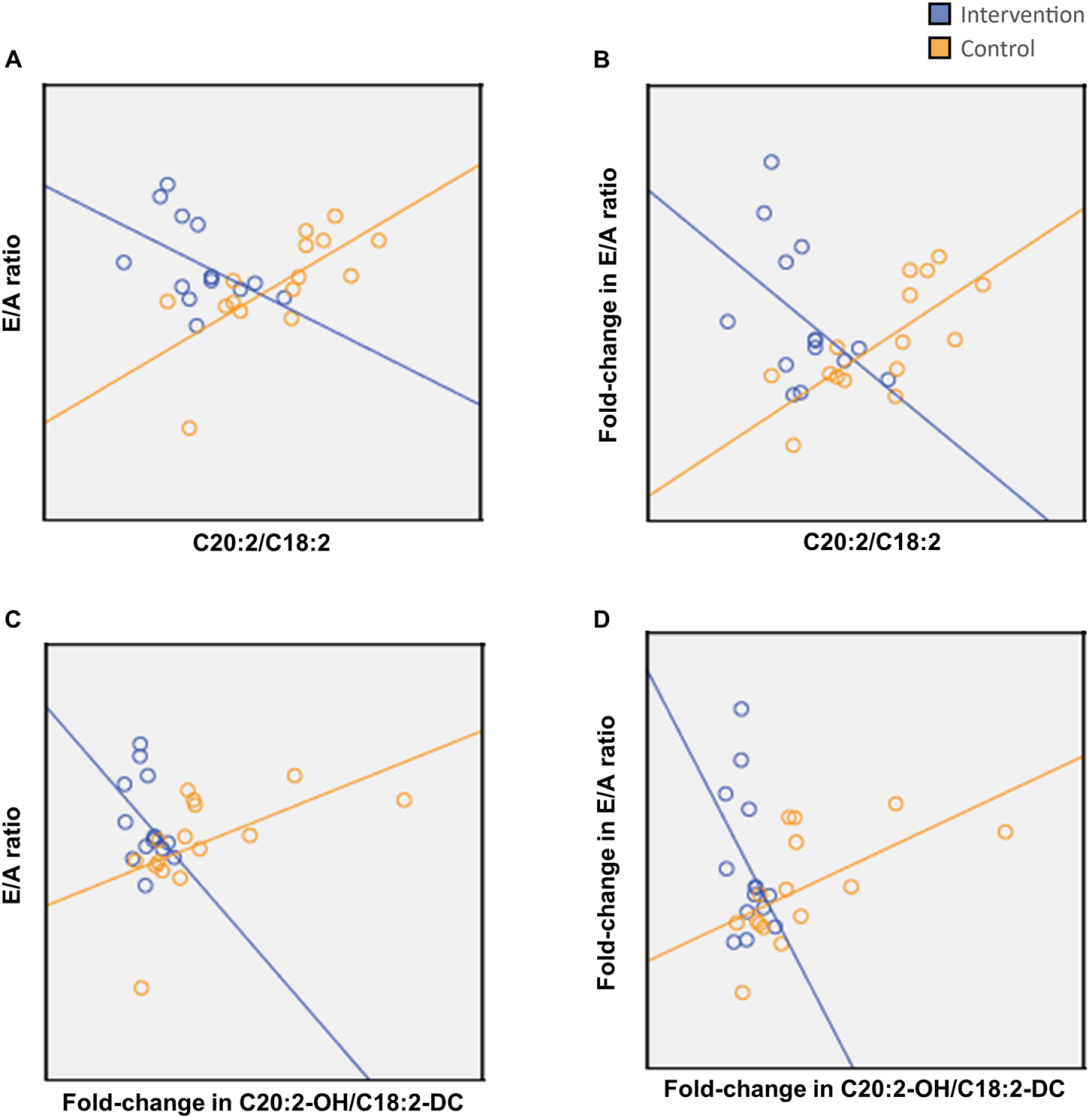
Correlations between serum long-chain acylcarnitines with E/A ratio. **A**, **B** Change in C20:2/C18:2 plotted against change in E/A ratio (**A**) and fold-change in E/A ratio (**B**). **C**, **D** Fold-change in C20:2-OH/C18:2-DC plotted against E/A ratio (**C**) and fold-change in E/A ratio (**D**). Spearman’s rank-order correlation was used to assess the relationships between metabolites and cardiac function. Adjustments for age were made using multiple regression. In the Intervention group (*n* = 14), inverse correlations between C20:2/C18:2 and E/A ratio (**A**, R = −0.535, *p* = 0.049, adj. *p* = 0.078) and E/A fold-change (**B**, R = −0.486, *p* = 0.078, adj. *p* = 0.084) were not significant; while in the Control group (*n* = 15), E/A ratio and E/A fold-change were both positively correlated (**A**, R = 0.795, *p* < 0.001, adj. *p* = 0.005; **B**, R = 0.789, *p* < 0.001, adj. *p* = 0.003 respectively). Comparing C20:2-OH/C18:2-DC, E/A ratio and E/A fold-change both trended towards an inverse correlation in the Intervention group (**C**, R = −0.361, age-adjusted *p* = 0.160; **D**, R = −0.358, adj. *p* = 0.159, respectively) while both having a positive correlation in the Control group (**C**, R = 0.742 age-adjusted *p* = 0.030; **D**, R = 0.736 adj. *p* = 0.017, respectively). E/A ratio indicates ratio of early to late peak diastolic mitral inflow velocity.

**Fig. 5:**
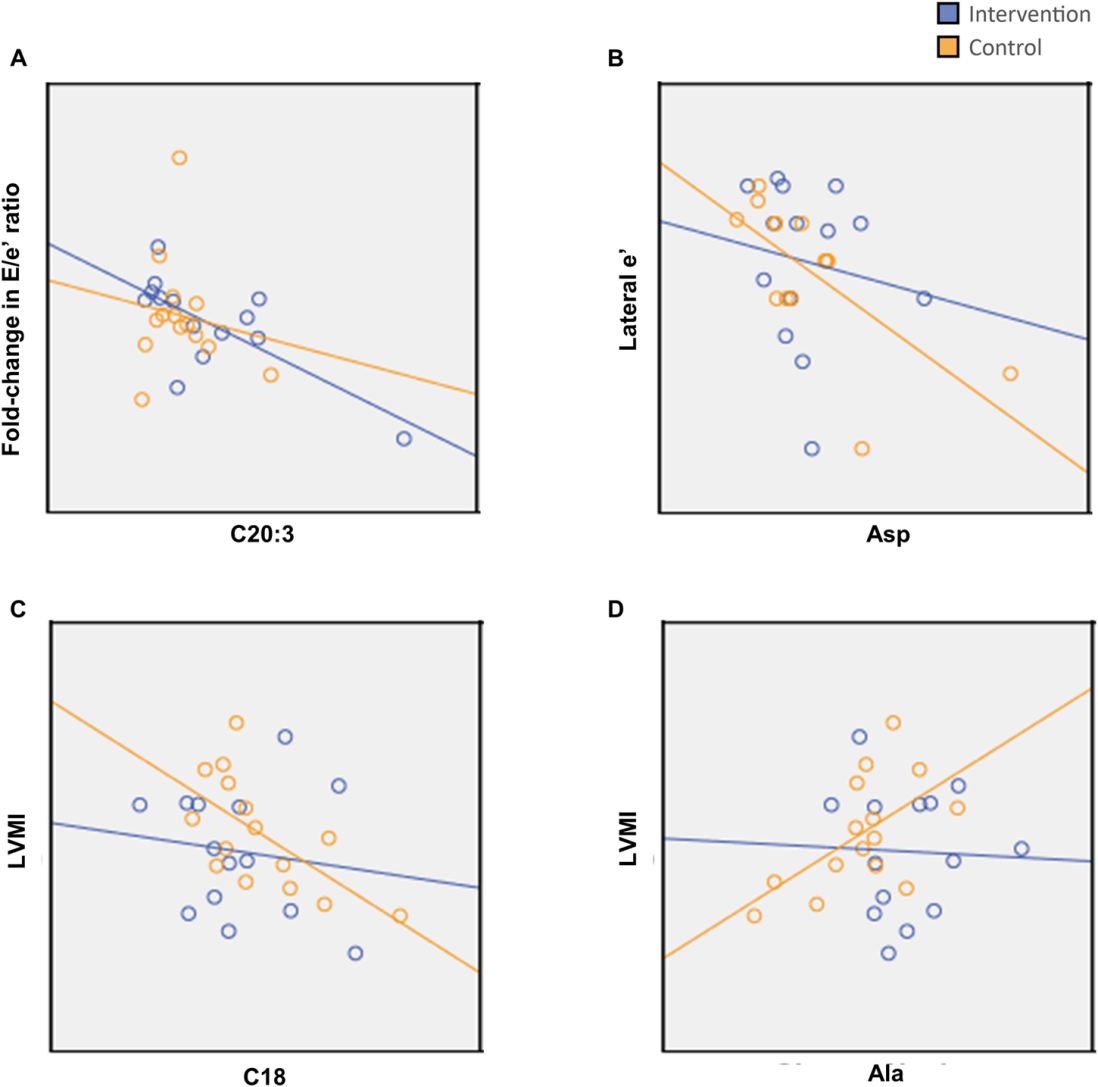
Correlations between Lateral e’, E/e' ratio, LVMI and serum metabolites. **A** change in C20:3 plotted against fold-change in E/e' ratio. **B** Change aspartate plotted against Lateral e’. **C**, **D** Change in a serum long-chain acylcarnitine C18 and alanine plotted against LVMI, respectively. Spearman’s correlation was used to assess the relationships between metabolites and cardiac function. Adjustments for age were made using multiple regression. **A** C20:3 was inversely correlated with E/e' ratio fold-change in the Intervention group (*n* = 14) (R = −0.653, *p* = 0.011, adj. *p* = 0.004) but not the Control group (*n* = 15) (R = −0.121, adj. *p* = 0.351). **B** Aspartate was not correlated with Lateral e' in the Intervention group (R = −0.311 adj. *p* = 0.477) but was negatively correlated in the Control group (R = −764, *p* = 0.001, adj. *p* = 0.016). **C,** **D** C18 and alanine were not correlated with LVMI in the Intervention group (**C**, R = −0.143, adj. p = 0.674; **D**, R = −0.029, adj. *p* = 0.874, respectively) but were positively correlated in the Control group (**C**, R = −0.614 *p* = 0.015, adj. *p* = 0.012; **D** R = 0.529, *p* = 0.043, adj. *p* = 0.018, respectively). Ala indicates Alanine; Asp, Aspartate; E/e’ ratio, ratio of early diastolic mitral inflow peak velocity (E) to lateral mitral annulus early diastolic peak velocity (e’); Lateral e’, lateral mitral annulus early diastolic peak velocity; LVMI left ventricular mass index.

Changes in several metabolites corresponded with changes in cardiac parameters. C20:2/C18:2 and C20:2-OH/C18:2-DC were positively correlated with the E/A ratio in the Control group but not the Intervention group (Fig. 4). Correlations between other long-chain acyl-carnitines and cardiac parameters were also observed, such as negative correlations between C20:3 with Lateral e’ in the Intervention group and positive correlations between C18 species with left ventricular mass index (LVMI) in the Control group (Fig. 5). For amino acids, aspartate negatively correlated with Lateral e’ while alanine positively correlated with LVMI in the Control group but not in the Intervention group (Fig. 5).

## Discussion

After twelve weeks of exercise training, we found alterations in serum metabolites corresponding to changes in cellular fuel oxidation and amino acid pathways that correlated with echocardiographic markers of diastolic function. The use of exercise to observe changes in serum metabolome in relation to cardiovascular parameters is a highlight of this work.

After twelve weeks of exercise, we observed reductions in levels of the long-chain acylcarnitine C20:20-OH/C18:2-DC in the Intervention group relative to Controls. Although no clinically relevant post-exercise changes in ventricular diastolic function were observed, non-cardiac mechanisms, such as skeletal muscle oxygen uptake and mitochondrial function suggested by circulating metabolites, may deepen mechanistic insights into exercise improvements in functional capacity^29^. Although C20:20-OH/C18:2-DC is a minor species of acylcarnitine, this finding suggests that alterations in fatty oxidation pathways occurred after the period of exercise. Accumulations in long-chain acylcarnitines (LCAC) are previously thought to have resulted from dysregulated fatty acid oxidation and mitochondrial insults and have been associated with impaired myocardial relaxation, preclinical heart failure, and predicted future myocardial dysfunction and major adverse cardiovascular events^15,30–32^. Previous studies have also found associations between LCACs with physical performance^33,34^. Positive associations between exercise and mitochondrial health also suggest biological plausibility^35,36^. Unlike short-chain acylcarnitines, long-chain acylcarnitines are derived exclusively from fatty acid metabolism. They are primarily metabolized in mitochondria, thus fulfilling their utility as a diagnostic tool for inborn fatty oxidation defects and a marker of mitochondrial dysfunction^37,38^. Other metabolomic studies have also identified evidence of skeletal mitochondrial dysfunction in patients HFpEF, reported by citrate synthase activity and porin expression, and biopsies of vastus lateralis muscles have also demonstrated decreased Type 1 oxidative fibres, which are dependent on aerobic respiration^39,40^. Incorporating these lines of evidence with our findings of reduced LCAC levels post-exercise, we postulate that exercise led to activations in mitochondrial pathways through metabolic switches at the proteomic or genomic levels, preventing the development of preclinical diastolic and vascular dysfunction observed in the Control group. These findings further support existing evidence that mitochondrial dysfunction in cardiac and skeletal muscles may be critical drivers of early cardiovascular aging, and exercise may reverse this^41–43^.

Corresponding relationships between serum metabolites and cardiac function in response to exercise intervention may shed some light on how exercise affects changes in the heart. Long-chain acylcarnitines (LCAC) C20:2, C20:2/C18:2, and C20:2-OH/C18:2-DC correlated with diastolic function and LV mass indices. This finding is unsurprising because LCACs are primarily metabolized in the heart and skeletal mitochondria. Thus, changes in plasma LCACs may reflect the activity of mitochondrial fatty oxidation in the heart and skeletal muscle^37,44^. We demonstrate this outside of a heart failure setting, suggesting a role to incorporate similar interventions that tackle upstream deteriorations in diastolic function among aging adults.

Besides acylcarnitines, there were also changes in amino acid levels, suggesting that the effect of exercise might be exerted through multiple metabolic pathways. Specifically, there was a trend towards higher arginine levels in the Intervention group. Arginine is involved in synthesizing other amino acids and compounds, including nitric oxide, which plays a crucial role in modulating vascular endothelial stiffness and left ventricular relaxation^45,46^. Declining levels of arginine have been associated with HFpEF^8,10,14^. However, arginine was not significant after adjusting for age.

While the Control group experienced decreases in Lateral e’ in and increases in markers of vascular stiffness, such as central diastolic blood pressure and pulse wave velocity, we did not find improvements in some other markers of LV diastolic function, such as the E/A ratio or E/e’ ratio. Age is an important determinant of vascular properties and diastolic function and could have contributed to observed differences between groups despite randomization. The differences in Lateral e’ and pulse wave velocity were non-significant after adjusting for age, indicating the possible influence of age. Central blood pressure was increased among Controls compared to baseline but were not different comparing post-Control with post-Exercise groups. Other randomized trials involving exercise interventions of longer durations have shown improvements in cardiovascular stiffness post-exercise, including central aortic stiffness (20 weeks) and invasive markers of LV myocardial stiffness (2 years)^47,48^. However, other study cohorts of shorter exercise duration have also not reported clear improvements in LV structure or function post-exercise^49,50^. In one such study, eight weeks of three sessions per week of supervised endurance and resistance training in patients post-myocardial infarction failed to show any significant improvement in Lateral e’ or E/e’ ratio^51^. A meta-analysis of eight RCTs of exercise training in HFpEF patients found that 12–24 weeks of exercise training improved functional tolerance without significant change in e’ or E/e’ ratio^49^. While increases in exercise frequency or duration may produce more observable changes in cardiac structure and function, our short-term exercise intervention was designed to capture changes in the metabolome within the defined period. Exercise trials in older patients have produced mixed-to-neutral effects on cardiac structure and function^52–54^. By identifying changes in the serum metabolome that were affected by exercise, our trial provides human molecular insights into the effect of exercise training, which may not be elucidated from gross cardiac functions.

There were several unexpected findings in our study. A medium-chain acylcarnitine (MCAC) (C12:2-OH/C10:2-DC) increased in the Intervention group but decreased in the Control group. Like LCAC, MCAC accumulation was thought to be associated with defects in fatty acid oxidation and mitochondrial dysfunction, such as the inadequate capacity of the tricarboxylic acid cycle (Krebs cycle) relative to acetyl-CoA generation^13,55^. Observational studies have linked MCACs to arterial stiffness and increased cardiovascular risk^13^. One possible explanation is that exercise increased fatty-acid oxidation and chain-shortening of long-chain acyl-carnitines, resulting in increased chain-shortened metabolites, including MCAC^55–57^. However, C12:2-OH/C10:2-DC was not significant after adjusting for age.

Another unexpected finding was the increase in alanine levels in the Intervention group relative to the Control group. Other observational studies have associated higher alanine levels with abnormal E/A function and HFpEF^8,10,14^. Alanine is an amino acid involved in several essential metabolic pathways, most notably in glucose and protein metabolism, such as nitrogen transport from peripheral tissues to the liver via the glucose-alanine cycle^58,59^. One possible explanation for the increase in alanine is an increase in demand for fuel metabolism post-exercise, resulting in higher amino acid catabolism to feed anaplerotic substrates and activation of hepatic glucose recovery pathways via gluconeogenesis in skeletal muscles^60^. However, alanine was not significant after adjusting for age.

There are several limitations to consider. Firstly, the marginal values in some of the observed changes may be underpowered due to the small study size. Despite the low power, these participants were from a low-risk community cohort for which observed relationships are likely to be underestimated rather than over-estimated. Nevertheless, this pilot study provides estimations of effect sizes for future trials in this field. Secondly, there was a female predominance in the study, which needs to be considered in future larger studies to account for sex dimorphisms in the serum metabolome. Differences in age between the Control and Exercise groups may also affect the interpretation of metabolomic, cardiac, and vascular data^61,62^. However, studies have shown that age and female sex are associated with greater ventricular stiffness in community adults without cardiovascular disease. Thus, our findings are relevant to real-world scenarios^1,6^. Thirdly, dietary, and other lifestyle factors pertinent to metabolomic perturbations were not adjusted, although randomization may correct inherent differences between groups. No restrictions on diet or exercise were prescribed during the intervention period, and the fasting state of blood tests was not specified in this real-world study of older adults^9^. While diet and exercise levels may affect metabolites in the blood^63^, fasting has not been a major source of variability in most metabolites although acylcarnitines may demonstrate some variability based on fasting status^64,65^. For future studies, correlations between hexoses and essential amino acids with markers of mitochondrial metabolism may provide insights into the nutritional status of participants^66^. Fourth, using targeted metabolomic profiling allowed for quantifying absolute metabolite concentrations but resulted in a limited breadth of analysis. However, targeted profiling allowed us to demonstrate a quantifiable extent of change post-exercise. Although these metabolites represent a small portion of the human metabolome, they report on critical pathways for cellular metabolism. Fifth, the trial involved moderately low-intensity exercise over a short duration, which might have led to null effects on skeletal muscle mass, a relevant variable of interest in aging studies^67^. Despite clinically indistinguishable changes in skeletal muscle mass, circulating LCAC and alanine levels may help track changes in metabolic pathways common to cardiac and skeletal muscle. Future studies that include biomarkers, such as muscle mass-derived cystatin C or nitric oxide-mediated epithelial signalling citrate/arginine ratio, may provide further mechanistic insights^68,69^. Sixth, while the lower prevalence of cardiovascular risk factors in this sample may limit generalizability, the results are less influenced by factors such as diabetes mellitus and hypertension, which may be differentially associated with branched-chain amino acids and other lipid pathways^70,71^. Finally, non-invasively obtained echocardiographic data to calculate stroke volume, cardiac output, arterial elastance, and arterial compliances are estimates and may not be accurate for interpretation compared to invasive angiographic data. However, it may be impractical to subject healthy patients to invasive angiography in clinical practice, and more helpful to rely on echocardiographic data.

Despite these limitations, our findings deepen our understanding of the mechanistic benefits of exercise in aging-associated cardiovascular disease. Further studies with larger cohorts or longer follow-ups may better depict the clinical impact of these mechanisms.

## Conclusion

Our pilot data demonstrated improvements in diastolic function after an exercise intervention with corresponding changes in cellular fuel oxidation and central carbon metabolism post-exercise. Corresponding relationships between serum metabolites and cardiac function were mapped out in response to exercise intervention. These findings highlight the potential for exercise strategies to target cellular fuel oxidation or central carbon metabolism pathways to concurrently impact the heart and related metabolic systems in prevention.

### Supplementary information

Supplementary Information

Description of Additional Supplementary Files

Supplementary Data 1

Reporting Summary

## Data Availability

*The individual participant data underlying this article are not publicly available and cannot be shared* outside of the study site unless explicit approval from the SingHealth Centralized Institutional Review Board, following an approved proposal by an independent review committee and a signed data access agreement; proposals should be directed to the corresponding author. *The source data for the figures are available as Supplementary Data*. T*he study protocol will be shared on reasonable request to the corresponding author immediately following publication*.
