# Supplementary material for: Effects of short-term moderate intensity exercise on the serum metabolome in older adults: a pilot randomized controlled trial
Source: Commun Med (Lond). 2024 May 4;4:80. doi: 10.1038/s43856-024-00507-w (PMC11069586; doi:10.1038/s43856-024-00507-w)
Supplement: Supplementary file 1 — Supplementary Information [file 43856_2024_507_MOESM1_ESM.pdf]

Effects of short-term moderate intensity exercise on the serum metabolome in older adults: a pilot randomized controlled trial.

Jie Jun Wong[1]; Jien Sze Ho[1,2]; Louis LY Teo[1,2]; Hai Ning Wee[2]; Kee Voon Chua[2]; Jianhong Ching[2]; Fei Gao[1,2]; Swee Yaw Tan[1,2]; Ru San Tan[1,2]; Jean-Paul Kovalik[2,3*]; Angela S. Koh[1,2*]

[1] National Heart Centre Singapore, Singapore, Singapore

[2] Duke-NUS Medical School, Singapore, Singapore

[3] Singapore General Hospital, Singapore, Singapore

* These authors jointly supervised this work

Corresponding author:

Angela S. Koh

National Heart Centre Singapore

5 Hospital Drive, Singapore 169609

Email: angela.koh.s.m@singhealth.com.sg

Telephone: +65 6704 8961

Fax: +65 6222 9258

Running title: Cardiac Trial of Exercise and Metabolomics

Word count (Abstract): 298

Word count (Main text): 3,556

Number of tables: 4

Number of figures: 4

Trial registration: ClinicalTrials.gov Identifier: NCT03617653

Supplementary Table 1: Comparison of echocardiographic parameters after the intervention period in the Control and Exercise groups.

	Control (n=15)	Intervention (n=14)	
	**Post**	**Post**	**p-value**
IVSd, cm	0.79 (0.11)	0.72 (0.08)	0.112
LVIDd, cm	4.37 (0.41)	4.57 (0.39)	0.451
LVIDs, cm	2.80 (0.3)	2.93 (0.42)	0.331
LVPWd, cm	0.71 (0.06)	0.75 (0.08)	0.123
LVPWs, cm	1.20 (0.19)	1.19 (0.17)	0.856
LVOT, cm	2.03 (0.08)	2.09 (0.14)	0.377
AO, cm	2.80 (0.27)	2.92 (0.52)	0.960
LA, cm	3.24 (0.64)	3.51 (0.45)	0.247
LVEF, %	64.73 (2.79)	63.14 (3.48)	0.252
LVFS, %	35.33 (6.40)	37.14 (7.26)	0.561
LV mass, g	100.7 (21.7)	106.0 (20.5)	0.780
LV mass index, g/m2	62.5 (12.2)	63.2 (7.8)	0.880
MV DT, ms	196.2 (21.2)	198.8 (27.6)	0.949
PASP, mmHg	21.3 (2.5)	21.2 (3.1)	0.979

Supplementary Table 1: Comparison of echocardiographic parameters after the intervention period in the Control and Exercise groups. Values are shown in mean (standard deviation). Mann Whitney U tests were used for comparisons. P-values are two-tailed. AO indicates aortic outflow tract diameter; IVSd, Interventricular septum end-diastolic diameter; LA, Left atrium diameter; LVEF, Left ventricular ejection fraction; LVFS, Left ventricular fractional shortening; LVIDd, Left ventricular internal diameter in diastole; LVIDs, Left ventricular internal diameter in systole; LVOT, Left ventricular outflow tract diameter; LVPWd, Left ventricular posterior wall thickness in diastole; LVPWs, Left ventricular posterior wall thickness in systole; MV DT, Mitral valve deceleration time; PASP, Pulmonary artery systolic pressure.

Supplementary Table 2. Age-adjusted change in Lateral e' (cm/s)

	**Age-adjusted mean difference (cm/s)**	**Unstandardized B coefficient**	**Standardized beta coefficient**	**Age-adjusted p-value**
Control group (n=15)	-1.41 (2.23)	-0.097	-0.16	0.585
Intervention group (n=14)	0.098 (2.55)	0.42	0.73	0.003

Supplementary Table 2. Age-adjusted change in Lateral e' (cm/s). Values are shown as mean (standard deviation). Lateral e’ changes before and after the study period in Control and Intervention groups were age-adjusted using multiple regression. P-values are two-sided. Lateral e’ indicates early diastolic lateral mitral annulus peak velocity.

Heatmap of Metabolite Changes

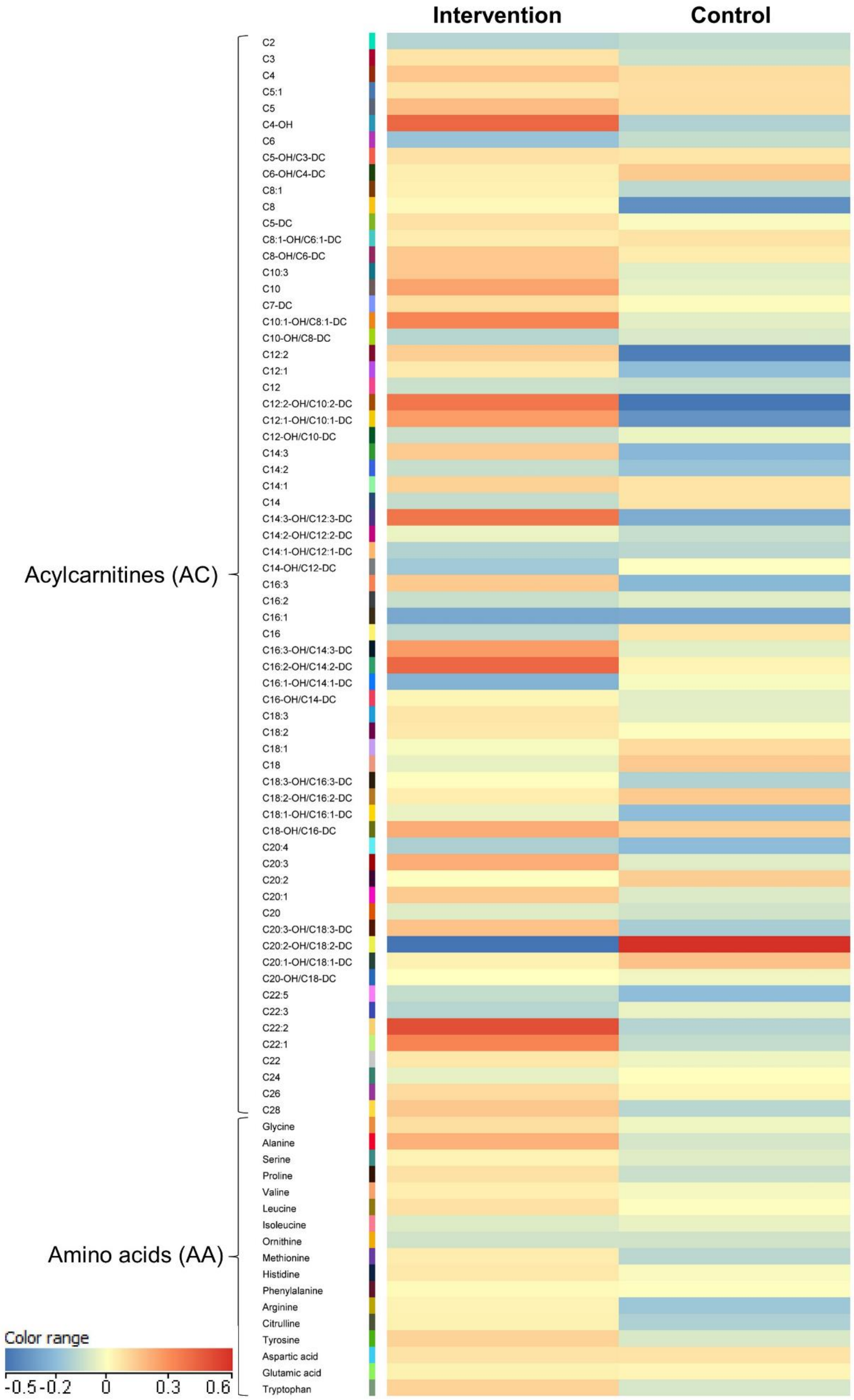

Supplementary Figure 1. Heatmap Showing Metabolite Changes. Serum samples were collected from subjects before and after the intervention. Acyl-carnitine and amino acids were determined for all samples. Measurements were taken from distinct samples. Post/pre fold-change was determined for each metabolite from every subject. The heatmap compares the average metabolite level for either no intervention or exercise to the average metabolite level for all subjects. Subjects are grouped along the y-axis according to Control (n=15) vs Intervention (n=14). Dark blue indicates strong reduction in metabolite level with intervention, Red indicates strong increase in metabolite with intervention.
