# Supplementary material for: Effects of short-term moderate intensity exercise on the serum metabolome in older adults: a pilot randomized controlled trial
Source: Commun Med (Lond). 2024 May 4;4:80. doi: 10.1038/s43856-024-00507-w (PMC11069586; doi:10.1038/s43856-024-00507-w)
Supplement: Supplementary file 2 — Description of Additional Supplementary Files [file 43856_2024_507_MOESM2_ESM.pdf]

Description of Additional Supplementary Files:

File name- Supplementary Data 1

File description- The source data for the figures are available as Supplementary Data
