# Supplementary material for: Effects of short-term moderate intensity exercise on the serum metabolome in older adults: a pilot randomized controlled trial
Source: Commun Med (Lond). 2024 May 4;4:80. doi: 10.1038/s43856-024-00507-w (PMC11069586; doi:10.1038/s43856-024-00507-w)
Supplement: Supplementary file 4 — Reporting Summary [file 43856_2024_507_MOESM4_ESM.pdf]

Corresponding author(s): Angela S. Koh
Last updated by author(s): Apr 21, 2024

Reporting Summary

Nature Portfolio wishes to improve the reproducibility of the work that we publish. This form provides structure for consistency and transparency in reporting. For further information on Nature Portfolio policies, see our Editorial Policies and the Editorial Policy Checklist.

Statistics

For all statistical analyses, confirm that the following items are present in the figure legend, table legend, main text, or Methods section.

n/a	Confirmed	
☐	☒	The exact sample size (n) for each experimental group/condition, given as a discrete number and unit of measurement
☐	☒	A statement on whether measurements were taken from distinct samples or whether the same sample was measured repeatedly
☐	☒	The statistical test(s) used AND whether they are one- or two-sided<br>*Only common tests should be described solely by name; describe more complex techniques in the Methods section.*
☐	☒	A description of all covariates tested
☐	☒	A description of any assumptions or corrections, such as tests of normality and adjustment for multiple comparisons
☐	☒	A full description of the statistical parameters including central tendency (e.g. means) or other basic estimates (e.g. regression coefficient) AND variation (e.g. standard deviation) or associated estimates of uncertainty (e.g. confidence intervals)
☐	☒	For null hypothesis testing, the test statistic (e.g. F, t, r) with confidence intervals, effect sizes, degrees of freedom and P value noted<br>*Give P values as exact values whenever suitable.*
☒	☐	For Bayesian analysis, information on the choice of priors and Markov chain Monte Carlo settings
☒	☐	For hierarchical and complex designs, identification of the appropriate level for tests and full reporting of outcomes
☐	☒	Estimates of effect sizes (e.g. Cohen's d, Pearson's r), indicating how they were calculated

Our web collection on statistics for biologists contains articles on many of the points above.

Software and code

Policy information about availability of computer code

Data collection	Metabolomics data acquisition and analysis were performed on an Agilent MassHunter Workstation B.06.00 Software.
Data analysis	Statistical analyses were done using IBM SPSS Statistics for Windows, Version 23 (IBM Corp, Armonk, NY, USA).

For manuscripts utilizing custom algorithms or software that are central to the research but not yet described in published literature, software must be made available to editors and reviewers. We strongly encourage code deposition in a community repository (e.g. GitHub). See the Nature Portfolio guidelines for submitting code & software for further information.

Data

Policy information about availability of data

All manuscripts must include a data availability statement. This statement should provide the following information, where applicable:

- Accession codes, unique identifiers, or web links for publicly available datasets
- A description of any restrictions on data availability
- For clinical datasets or third party data, please ensure that the statement adheres to our policy

The individual participant data underlying this article are publicly unavailable and cannot be shared outside of the study site unless explicit approval from the SingHealth Centralized Institutional Review Board, following an approved proposal by an independent review committee and a signed data access agreement; proposals should be directed to the corresponding author. The source data for the figures are available as Supplementary Data. The study protocol will be shared on reasonable request to the corresponding author immediately following publication.

Human research participants

Policy information about studies involving human research participants and Sex and Gender in Research.

Reporting on sex and gender	Our study findings were not restricted by sex. Sex was determined based on self-reporting. Randomization by sex-stratification was performed to ensure an equal distribution of sexes in each Intervention and Control arms. Of 29 participants, there were 5 males and 24 females in this study. There were no significant differences in sexes between the groups.
Population characteristics	Thirty community adults with no prior evidence of cardiovascular disease underwent transthoracic echocardiography and serum metabolomics sampling. They were randomized by sex stratification into Control (54.8 years ±3.55, males 13.3%) and Intervention groups (51.4 years ±4.34, males 20%). The Intervention group was younger by 3.2 years on average (p=0.037); otherwise, both groups exhibited similar clinical characteristics, including body mass index, waist circumference, blood pressure, resting pulse rate, and maximal oxygen uptake. There were no participants with hypertension or diabetes mellitus. One participant in the Intervention group had dyslipidaemia.
Recruitment	Community adults with no prior evidence of cardiovascular disease were recruited from the Cardiac Aging Study. Thirty participants volunteered to participate in this exercise trial between February 2019 and December 2019. Participants were recruited if they were 40 and above, were willing and able to provide informed consent, and had at least grade 1 diastolic dysfunction on echocardiography based on the latest guidelines. Participants with a history of cardiovascular disease, stroke, cancer, uncontrolled hypertension (i.e., systolic blood pressure ≥160 mmHg and/or diastolic blood pressure ≥90 mmHg despite being on treatment for hypertension), low blood pressure (i.e., systolic blood pressure <90mmHg or diastolic blood pressure <40mmHg), acute pulmonary embolus or pulmonary infarction, acute myocarditis or pericarditis, suspected or known dissecting aneurysm, acute systemic infection, or uncontrolled metabolic disease (e.g., diabetes, thyrotoxicosis or myxoedema) were excluded. Patients with neuromuscular, musculoskeletal, or rheumatoid disorders that are exacerbated by exercise were also excluded. Written informed consent was obtained from participants upon enrolment.
Ethics oversight	The institutional review board (CIRB/2018/2118) approved the study protocol.

Note that full information on the approval of the study protocol must also be provided in the manuscript.

Field-specific reporting

Please select the one below that is the best fit for your research. If you are not sure, read the appropriate sections before making your selection.

☒ Life sciences ☐ Behavioural & social sciences ☐ Ecological, evolutionary & environmental sciences

For a reference copy of the document with all sections, see nature.com/documents/nr-reporting-summary-flat.pdf

Life sciences study design

All studies must disclose on these points even when the disclosure is negative.

Sample size	Thirty participants were recruited in this pilot study.
Data exclusions	Exclusion criteria were pre-specified. Participants with a history of cardiovascular disease, stroke, cancer, uncontrolled hypertension (i.e., systolic blood pressure ≥160 mmHg and/or diastolic blood pressure ≥90 mmHg despite being on treatment for hypertension), low blood pressure (i.e., systolic blood pressure <90mmHg or diastolic blood pressure <40mmHg), acute pulmonary embolus or pulmonary infarction, acute myocarditis or pericarditis, suspected or known dissecting aneurysm, acute systemic infection, or uncontrolled metabolic disease (e.g., diabetes, thyrotoxicosis or myxoedema) were excluded. Patients with neuromuscular, musculoskeletal, or rheumatoid disorders that are exacerbated by exercise were also excluded.
Replication	As this study is a prospective randomized control trial, findings were not replicated.
Randomization	Participants were randomized using sex-stratification to ensure equal distribution of sexes between Intervention and Control arms.
Blinding	Treadmill test results were read by independent board-certified cardiologists blinded to participation details. Echocardiograms were performed by ultrasonographers blinded to the allocation of groups and interpreted by independent board-certified cardiologists blinded to participation details.

Reporting for specific materials, systems and methods

We require information from authors about some types of materials, experimental systems and methods used in many studies. Here, indicate whether each material, system or method listed is relevant to your study. If you are not sure if a list item applies to your research, read the appropriate section before selecting a response.

Materials & experimental systems

n/a	Involved in the study
☒	☐ Antibodies
☒	☐ Eukaryotic cell lines
☒	☐ Palaeontology and archaeology
☒	☐ Animals and other organisms
☐	☒ Clinical data
☒	☐ Dual use research of concern

Methods

n/a	Involved in the study
☒	☐ ChIP-seq
☒	☐ Flow cytometry
☒	☐ MRI-based neuroimaging

Clinical data

Policy information about clinical studies

All manuscripts should comply with the ICMJE guidelines for publication of clinical research and a completed CONSORT checklist must be included with all submissions.

Clinical trial registration: NCT03617653

Study protocol: https://classic.clinicaltrials.gov/ct2/show/NCT03617653

Data collection: Community adults with no prior evidence of cardiovascular disease underwent transthoracic echocardiography as part of the Cardiac Aging Study, a prospective study that examines characteristics and determinants of cardiac structural and functional changes at age. Thirty participants volunteered to participate in this exercise trial between February 2019 and December 2019. Participants were recruited if they were 40 and above, were willing and able to provide informed consent, and had at least grade 1 diastolic dysfunction on echocardiography based on the latest guidelines. Participants with a history of cardiovascular disease, stroke, cancer, uncontrolled hypertension (i.e., systolic blood pressure ≥160 mmHg and/or diastolic blood pressure ≥90 mmHg despite being on treatment for hypertension), low blood pressure (i.e., systolic blood pressure <90mmHg or diastolic blood pressure <40mmHg), acute pulmonary embolus or pulmonary infarction, acute myocarditis or pericarditis, suspected or known dissecting aneurysm, acute systemic infection, or uncontrolled metabolic disease (e.g., diabetes, thyrotoxicosis or myxoedema) were excluded. Patients with neuromuscular, musculoskeletal, or rheumatoid disorders that are exacerbated by exercise were also excluded. Written informed consent was obtained from participants upon enrolment.

Outcomes: The study's primary outcome measure was the change in metabolomic markers after 12 weeks of exercise intervention. Post-hoc analyses were carried out to examine associations between metabolomic markers, echocardiographic left ventricle indices, and vascular compliance at baseline and with respect to their change over time.
